# Alpha-oxoglutarate inhibits the proliferation of immortalized normal bladder epithelial cells via an epigenetic switch involving ARID1A

**DOI:** 10.1038/s41598-018-22771-2

**Published:** 2018-03-14

**Authors:** Muhammad Shahid, Nicole Gull, Austin Yeon, Eunho Cho, Jooeun Bae, Hyun Seok Yoon, Sungyong You, Hana Yoon, Minjung Kim, Benjamin P. Berman, Jayoung Kim

**Affiliations:** 1 Departments of Surgery and Biomedical Sciences, Cedars-Sinai Medical Center, Los Angeles, CA USA; 2 Center for Bioinformatics and Functional Genomics, Department of Biomedical Sciences, Cedars-Sinai Medical Center, Los Angeles, California, USA; 3 University of California Los Angeles, Los Angeles, CA USA; 4 Department of Urology, School of Medicine, Ewha Womans University, Seoul, Republic of Korea; 5 Department of Molecular Oncology, Moffitt Cancer Center, Tampa, Florida USA; 6 Samuel Oschin Comprehensive Cancer Institute, Cedars-Sinai Medical Center, Los Angeles, CA USA; 7 Department of Urology, Ga Cheon University College of Medicine, Incheon, Republic of Korea

## Abstract

Interstitial cystitis (IC) is a chronic urinary tract disease that is characterized by unpleasant sensations, such as persistent pelvic pain, in the absence of infection or other identifiable causes. We previously performed comprehensive metabolomics profiling of urine samples from IC patients using nuclear magnetic resonance and gas-chromatography/mass spectrometry and found that urinary α-oxoglutarate (α-OG), was significantly elevated. α-OG, a tricarboxylic acid (TCA) cycle intermediate, reportedly functions to suppress the proliferation of immortalized normal human bladder epithelial cells. Here, we identified AT-rich interactive domain 1 A (ARID1A), a key chromatin remodeler, as being hypomethylated and upregulated by α-OG treatment. This was done through EPIC DNA methylation profiling and subsequent biochemical approaches, including quantitative RT-PCR and western blot analyses. Furthermore, we found that α-OG almost completely suppresses ten-eleven translocation (TET) activity, but does not affect DNA methyltransferase (DNMT) activity. Altogether, our studies reveal the potential role of α-OG in epigenetic remodeling through its effects on ARID1A and TET expression in the bladder. This may provide a new possible therapeutic strategy in treating IC.

## Introduction

Urine is a critical biological fluid that is filtered through the kidneys and stored in the bladder. It contains the expression of many metabolites, such as urea (from amino acid metabolism), inorganic salts (chloride, sodium, and potassium), creatinine, ammonia, organic acids, various water-soluble toxins, and pigmented products from hemoglobin breakdown. Because urination is also the primary route through which the body eliminates water-soluble waste and extra unnecessary products, urine has long been considered as an expendable composite. However, more recently, urine has been acknowledged as an uninvestigated biomarker source with great potential use for disease diagnosis. While previous studies of urine have mainly focused on its chemical composition, new focus is being placed on its metabolic properties as indicative sources for medical disorders. Although the complexity of sources within metabolites creates many obstacles in urine analysis, progress in the field has been promising. Urine analysis could prove to be tremendously beneficial^1^.

Interstitial cystitis/bladder pain syndrome (IC/BPS, hereafter IC) is a debilitating urological dysfunction that presents itself as a constellation of symptoms, including bladder pain, urinary urgency, frequency, nocturia, and small voided volumes, in the absence of other identifiable etiologies^2–7^. The prevalence of IC in the United States is 3–6% in women and 2–4% in men^8,9^. IC patients experience substantial decline in physical activity, social interaction, and quality of life, mainly due to chronic pelvic pain, frequent urination, and other symptoms^10–12^. Despite the considerable burden of IC on public health, there is no diagnostic gold standard for IC. Urologists currently use the American Urological Association guidelines for diagnosing IC in the absence of other identifiable causes for symptoms (e.g., urinary tract infection and cancer). Therefore, clearer disease indicators and studies involving larger populations are needed to further our understanding of the molecular and cellular mechanisms behind IC. Current treatment options also remain suboptimal. Because so little is known regarding IC, palliative care is the only option available to patients.

In an effort to identify IC biomarkers, we have been keen on examining urine as a bio-resource; mainly because bodily fluid most proximal to a disease site provides a wealth of informative biomarkers^13–15^. A series of our studies involving metabolic profiling revealed a list of candidate urinary biomarkers associated with IC. Previously, we used nuclear magnetic resonance (NMR) and liquid chromatography−mass spectrometry (LC−MS) to show substantial differences between the metabolic profiles of urine from IC patients and those of healthy control subjects^16^. We found that α-oxoglutarate (α-OG) was significantly increased in IC patients. α-OG, also known as α-ketoglutarate, is a key Krebs cycle intermediate and regulator of cellular redox states^17^. More recently, α-OG has been reported to function in epigenetic regulation, contributing to metabolic reprogramming and macrophage activation^17,18^. Treatment with α-OG in bladder epithelial cells suppressed cell proliferation and was consistent with previous observations by other groups that found α-OG inhibited cell cycle transitions through the up or downregulation of p21^Waf1/Cip1^, p27^Kip1^, cyclin D1, and Rb. However, no functional impacts or mechanisms of urinary α-OG have been proposed in the setting of bladder wall abnormalities or diseases and no correlations have been previously described.

In this study, we examined the biological impact of increased levels of α-OG. Given that our previous global metabolomics profiling of IC patient urine suggested the possibility of finding non-invasive metabolic signatures, this study focused on gaining new insight into the mechanism of IC through investigating the biological meaning of upregulated α-OG. Here, our Infinium MethylationEPIC profiling revealed the bladder DNA methylome as being responsive to α-OG. Our findings provide evidence suggesting that α-OG may change the physiology of bladder epithelial cells at the epigenetic and metabolic levels.

## Materials and Methods

### Cell Culture and Proliferation Assay

Immortalized normal human bladder epithelial cells, TRT-HU1, were maintained as described previously^19^. The TRT-HU1 cell line was constructed and extensively characterized in previously published papers. Cells were cultured in Dulbecco’s modified Eagle’s medium (DMEM), containing 10% fetal bovine serum (FBS, Invitrogen), 1% penicillin/streptomycin, and 1% L-glutamine (Sigma, St. Louis, MO) in a 37 °C humidified incubator with 5% CO_2_. TRT-HU1 cells were seeded in 10 cm culture plates at a density of 1 × 10^6^ cells in standard growth medium. When the cells reached approximately 80% confluence, they were treated and incubated with 10 mM of α-OG or vehicle for 72 h. For the crystal violet staining cell proliferation assay, cells were stained with 0.5% crystal violet (Sigma-Aldrich Corp., St. Louis, MO, USA) in 30% ethanol for 10 minutes at room temperature. The cells were lysed in a 1% SDS solution. The absorbance of the solution was measured using a microplate spectrophotometer at a wavelength of 595 nm.

### Epigenome-wide DNA methylation

Methylation profiles were obtained using the Illumina Infinium HumanMethylationEPIC BeadChip kit, which assayed approximately 850,000 methylation sites per sample at a single nucleotide resolution^20^. Methylation scores were computed as β-values, which took into account the ratio of methylated probe intensities and the overall probe intensities (methylated and un-methylated plus a constant, C = 100). β-values are between 0 and 1. Assays and validations were performed according to the manufacturer’s recommendations.

### Bioinformatic analysis

A data analysis pipeline was established using a combination of R Studio and Bioconductor. Illumina HumanMethylationEPIC data (IDAT files) were imported into the Bionconductor minfi package. Extension of minifi package to support EPIC arrays is described in previous literature^21^. Background in the data was corrected using PreprocessNoob^22^. No normalization was done. Data was mapped to the genome according to standards outlined by minfi. Annotations were added using IlluminaMethylationManifest, a class built within the minfi package. Volcano plots were constructed using the Biconductor package, TCGAbiolinks^23^. Heatmaps were created using the Bioconductor package, ComplexHeatmap^24^. Differentially methylated genes (DMGs) were identified using a simple T-test comparing treated and un-treated groups. Additionally, we compared the mean β-values of two groups and only included probes that had a difference of 10%. Using the DAVID software (Ver. 6.8)^25^, we performed functional enrichment analysis of the DMGs to identify cellular processes and pathways perturbed by α-OG treatment.

### Network analysis

From the functional enrichment analysis, we identified the hypo-methylated genes as those involved in cellular processes, including cell-cell adhesion and chromatin remodeling. We then searched for interactions among the hypo-methylated genes using the STRING database^26^. A network model was reconstructed with the interactions between these genes and visualized using Cytoscape (Ver. 3.4)^27^. Node size represents degree centrality, which is the number of interactions in the network model, and edge thickness represents interaction scores, which are combined scores obtained from the STRING database^26^. These combined scores were computed by adding the probabilities from different evidence of interactions in the database and correcting for the probability of randomly observing an interaction.

### Quantitative RT-PCR

Total RNA was purified using the MagNA Pure Compact RNA isolation kit, according to the manufacturer’s instructions (Roche). cDNA was synthesized using the iScript cDNA Synthesis Kit (Bio-Rad). Q-PCR was carried out with iTaq universal SYBR green supermix (BioRad) on a 7500 Fast Real-Time PCR system (Life Technologies). The annealing temperature for the qPCR was set to 60 °C and the total cycle number was 40. Actb was used as internal control for gene expression normalization. Primers used for qRT-PCR assay are described in Supplementary Table 1.

### Western blot analysis

Total cell lysates were prepared in lysis buffer (Bio-Rad) with protease inhibitors (Thermo Fisher). Protein levels were measured and 25 μg of cell lysates per condition were used for SDS-PAGE gel running. After transferring proteins to nitrocellulose membranes, western blot analysis was performed using antibodies specific for p53 (1:1000; sc-126; Santa Cruz), p21 (1:1000; 2947;Cell Signaling Technology), JunB (1:1000; 3746; Cell Signaling Technology), ARID1A (1:1000; 12354; Cell Signaling Technology), HDAC4 (1:1000; 5392; Cell Signaling Technology), PKD (1:1000; 2052; Cell Signaling Technology), β-catenin (1:1000; 8480; Cell Signaling Technology), PBRM1 (1:1000; 91894; Cell Signaling Technology), SMARCA2 (1:1000; 11966; Cell Signaling Technology), and β-actin (1:2000; A1978; Sigma-Aldrich). These were incubated overnight and followed by anti-specie-specific HRP-conjugated secondary antibodies (1:5000; 7074, 7076; Cell Signaling Technology) and then chemiluminescent detection. Some blots were stripped using Restore (Thermo Fisher) and detected using other primary antibodies.

### DNA Methyltransferase (DNMT) and ten-eleven translation (TET) activity assay

Cells were seeded 24 h before experiments and incubated with α-OG-containing media for 20 h. A commercially available EpiQuik™ DNMT Activity/Inhibition Assay Ultra Kit (Epigentek, Farmingdale) was used to determine DNMT enzymatic activity as per the manufacturer’s instruction. For the TET activity assay, the Epigenase^TM^ 5mC-Hydroxylase TET Activity/Inhibition Assay Kit (Colorimetric) was used, as per the manufacturer’s instructions. This sandwich-based 5hmC ELISA reads the conversion of methylated products to hydroxymethylated products through the presence of TET enzymes in samples.

### Ethics statement

The Ethics Committee of Ewha Womans University Hospital (Seoul, Republic of Korea) approved this study. The Institutional Review Board of Ewha University Hospital approved collection, curation and analysis of all samples. All subjects participated in this study provided written informed consent, and all experiments were performed in accordance with relevant guidelines and regulations.

### Subjects and biopsy tissue collection

Subjects, including IC patients (mean age, 52.5 ± 10.1) and healthy controls (mean age, 54.2 ± 9.3), were Asian-descent female residents in South Korea who were enrolled at their initial evaluation, before any treatments or procedures. They were diagnosed and recruited by two independent urologists, Drs. Hana Yoon and Hyun Seok Yoon, who have extensive clinical expertise in IC at an outpatient urology clinic at Ewha Womans University Hospital. Careful clinical diagnosis was done using clinical criteria in the AUA guidelines^28,29^; “An unpleasant sensation (pain, pressure, discomfort) perceived to be related to the urinary bladder, associated with lower urinary tract symptom(s) of more than 6 weeks duration, in the absence of infection or other identifiable causes” (http://www.auanet.org/education/guidelines/ic-bladder-pain-syndrome.cfm). Diagnostic criteria included persistent or recurrent chronic pelvic pain, pressure or discomfort perceived to be related to the urinary bladder accompanied by at least one other urinary symptom such as an urgent need to void or urinary frequency in the absence of any identifiable pathology. Work-up included symptom assessment, cystoscopic evaluation, physical examination, urodynamics, and/or urine culture. Patients with a history of other diseases (such as any types of cancer, inflammation, or diabetes, etc.) were excluded. Patients who were under any active treatment or medication were clearly excluded. Only subjects >2 months “free of treatment, therapy, or any medications”, such as hormone use, pentosan polysulphate, or intravesical instillation therapy, were included. Staging was done based on symptoms, bladder pain, and quality of life. Matched controls were carefully selected considering age, menopausal status, hormone use *et al*. Bladder biopsies were performed before procedures such as hydrodistention or fulguration. Biopsy sites included lateral walls, dome, posterior wall of bladder wall, but not from trigone, for both IC patients and controls.

### ARID1A immunohistochemistry (IHC) and scoring system

IHC analysis for ARID1A expression was performed on 5-μm slides using a polyclonal rabbit anti-ARID1A antibody (1:250, HPA005456; Sigma-Aldrich). Antigen retrieval was performed by submerging the tissue sections in citrate buffer (pH 6.0) and then in a steamer for 10 minutes. The sections were then incubated with the rabbit antibody at a dilution of 1:200 at 4 °C overnight. A positive reaction was detected by the EnVision + System (Dako).

### Statistics

All data were generated from experiments performed at least three times and expressed as mean ± standard deviation (SD). The student’s *t*-tests were used to determine the statistical significance of differences between samples treated under different conditions. Differences were considered statistically significant when p < 0.05 (*), p < 0.01 (**), or p < 0.001(***).

## Results

### α-OG, an IC-associated metabolite, suppresses cell proliferation in immortalized normal human bladder-derived epithelial cells

**Figure 1:**
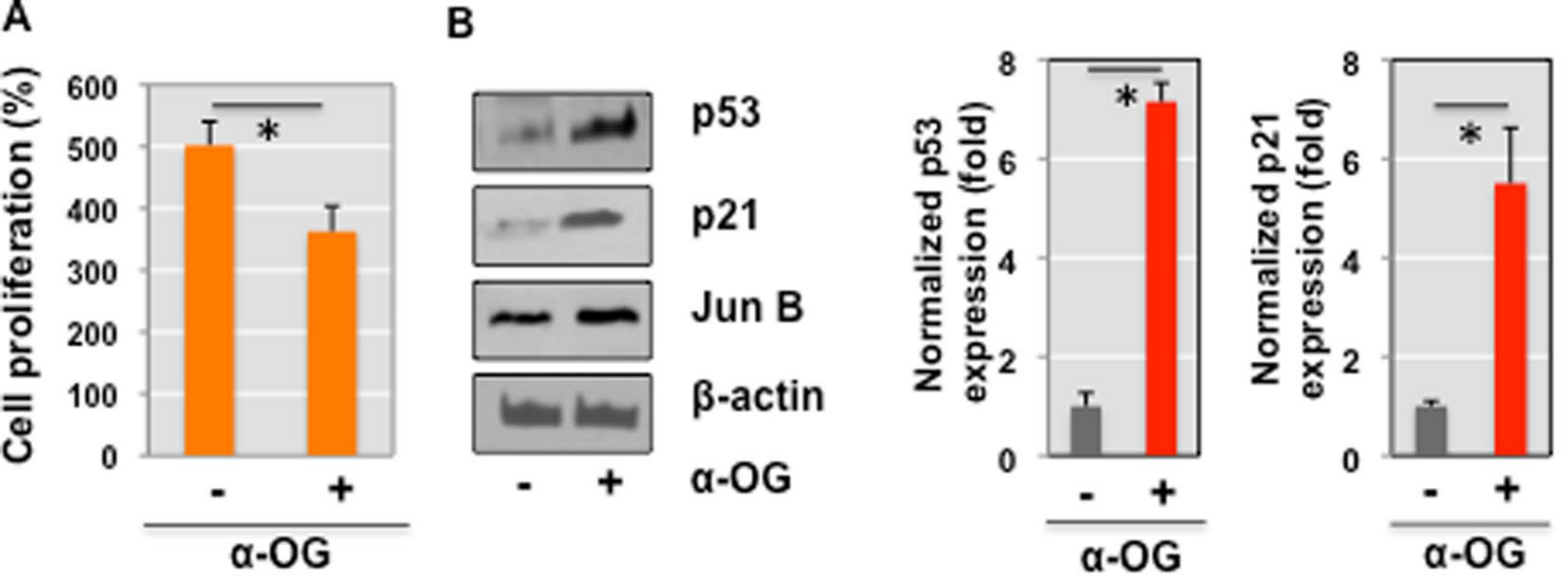
Cell proliferation was suppressed in response to α-OG treatment. (**A**) TRT-HU1, immortalized normal bladder epithelial cells were treated with 10 mM of α-OG for 72 h and then proliferation assay was performed as described in Methods. *P < 0.05 (two-sided Student’s t-test) compared with the control group. (**B**) Representative western blot showed the reduced levels of p53 and p21 expression in TRT-HU1 cells treated with α-OG. β-actin was used for protein normalization.

In light of our previous global metabolomics data linking α-OG to cell cycle arrest and proliferation suppression^16^, we first investigated the effects of α-OG in immortalized normal bladder epithelial cells. Cell cycle transition and proliferation were measured in TRT-HU1 cells treated with or without 10 mM of α-OG. Consistent with previous reports, we observed significant suppression of cell proliferation in response to α-OG **(**Fig. 1A**)**. Western blot analysis was performed to ascertain the involvement of several key cell cycle and proliferation regulators. We found that levels of p53 and p21 significantly increased while levels of JunB modestly increased in the presence of α-OG **(**Fig. 1B).

### α-OG perturbed the epigenetic architecture of immortalized normal bladder epithelial cells

**Figure 2:**
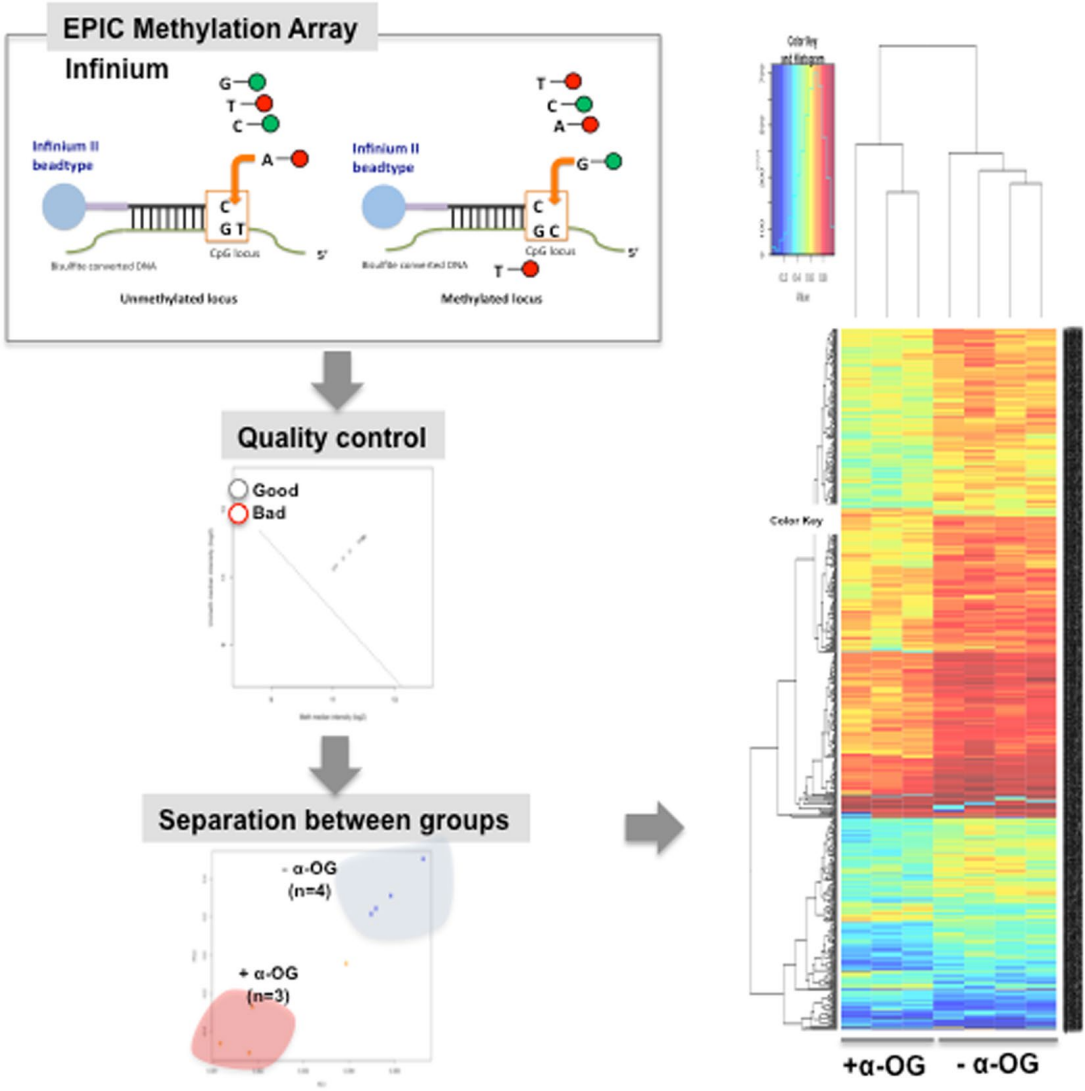
Experimental scheme describing EPIC DNA methylation profiling and the following bioinformatics analysis.

Given previous reports demonstrating that α-OG modulates epigenetic regulation, we next tested our hypothesis that the global DNA methylome is altered in response to α-OG in immortalized normal human bladder epithelial cells^18^. In order to assess whether the DNA methylome is changed by α-OG, EPIC array was performed. Our workflow is described in Fig. 2. Our analysis and data requisition resulted in differentially methylated genes (DMGs) in response to α-OG (Supplementary Table 2–4).

**Figure 3:**
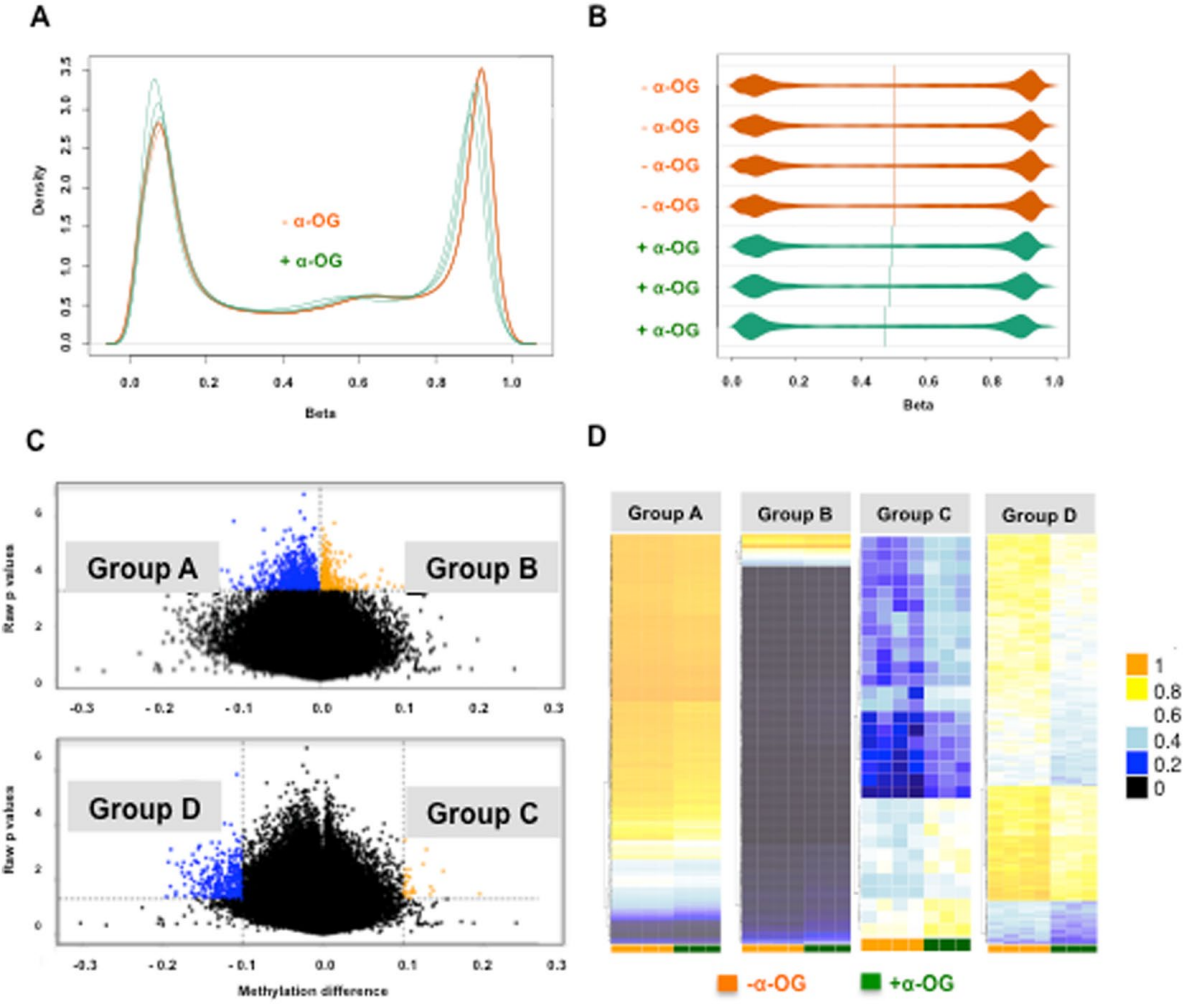
Reprogrammed DNA methylation architecture by α-OG treatment. (**A**) QC (density plots) of probes for DNA methylation levels (β-values). The orange group represents untreated samples, which show a slight hypermethylation pattern. The green group, α-OG-treated samples, shows a more even distribution of beta values across probes. **(B)** Density Bean Plot of probes for β-values in two experimental samples. Distribution of β-values across each sample (orange, untreated; green, α-OG-treated) was shown. **(C)** Volcano Plots indicates differentially methylated probes between untreated and treated samples. Each dot represents a single CpG probe on the array, with hypomethylated probes colored blue and hypermethylated probes colored orange. The upper plot defines differential methylation based on statistical significance alone (Group A – hypomethylated probes: p < 0.0005, Group B – hypermethylated probes: p < 0.0005). The lower plot shows the exact same volcano plot, but defines differential methylation based on both statistical significance and β-value difference (Group C - hypermethylated probes: p < 0.05 and β-value difference > 0.1, Group D - hypomethylated probes: p < 0.05 and β-value difference > 0.1). **(D)** Sample-specific β-values are shown as heatmaps for the probes in Groups A-D. Group A: 873 probes, Group B: 382 probes, Group C: 32 probes, Group D: 366 probes.

Using the results from the quality control (QC) test, density plots for probes were constructed for DNA methylation levels (β-values) in control and α-OG treated conditions. Untreated control samples (in orange) showed a slight hypermethylation pattern. The α-OG-treated samples (in green) showed a more even distribution of β values across probes (Fig. 3A). Density bean plots for probes **(**Fig. 3B**)** shows the DNA methylation levels (β-values) among different samples.

Following the QC steps within minfi (Fig. 3A, B), methylation data for 836,329 CpGs were analyzed. Because we were unable to use minfi’s stringent cutoffs to identify Differentially methylated regions (DMRs) with BumpHunter and dmpFinder, we opted to do our analysis using quantitative cutoffs, splitting into four groups of probes. The resulting volcano plots show the differentially methylated probes present between α-OG treatment and control conditions. Significantly hyper-methylated probes are shown in orange, while hypo-methylated probes are shown in blue. First, the most statistically robust CpGs were selected using a p-value cutoff of 0.0005. Unexpectedly, the most significant probes had the smallest differences in methylation β-values (β < 0.1). These two cutoffs yielded two groups, Group A and B, shown in Fig. 3C, as having 873 and 382 probes, respectively. The other two groups (C and D) were identified by looking at the largest difference in β-values present, β > 0.1. These probes were less statistically significant, with a p-value < 0.05. Group C only had 32 probes while Group D had 366. Overall, our analysis of probes that were most statistically significant and had the greatest magnitude of change showed a general shift towards increased global hypo-methylation. Based on these results, we opted to further analyze the groups that had the largest changes in β-values, focusing mostly on group D due to the limited number of probes in group C.

Gene tables for the groups with the largest numbers of identified probes, group A, B, and D, can be found in Supplementary Tables 2, 3, and 4, respectively). Heatmaps of all four groups of probes are shown in Fig. 3D. The heatmaps demonstrate a trend of increased hypo-methylation in probes of cells treated with α-OG, particularly in Group D.

### Biological meanings of DMGs responsive to α-oxoglutarate

**Figure 4:**
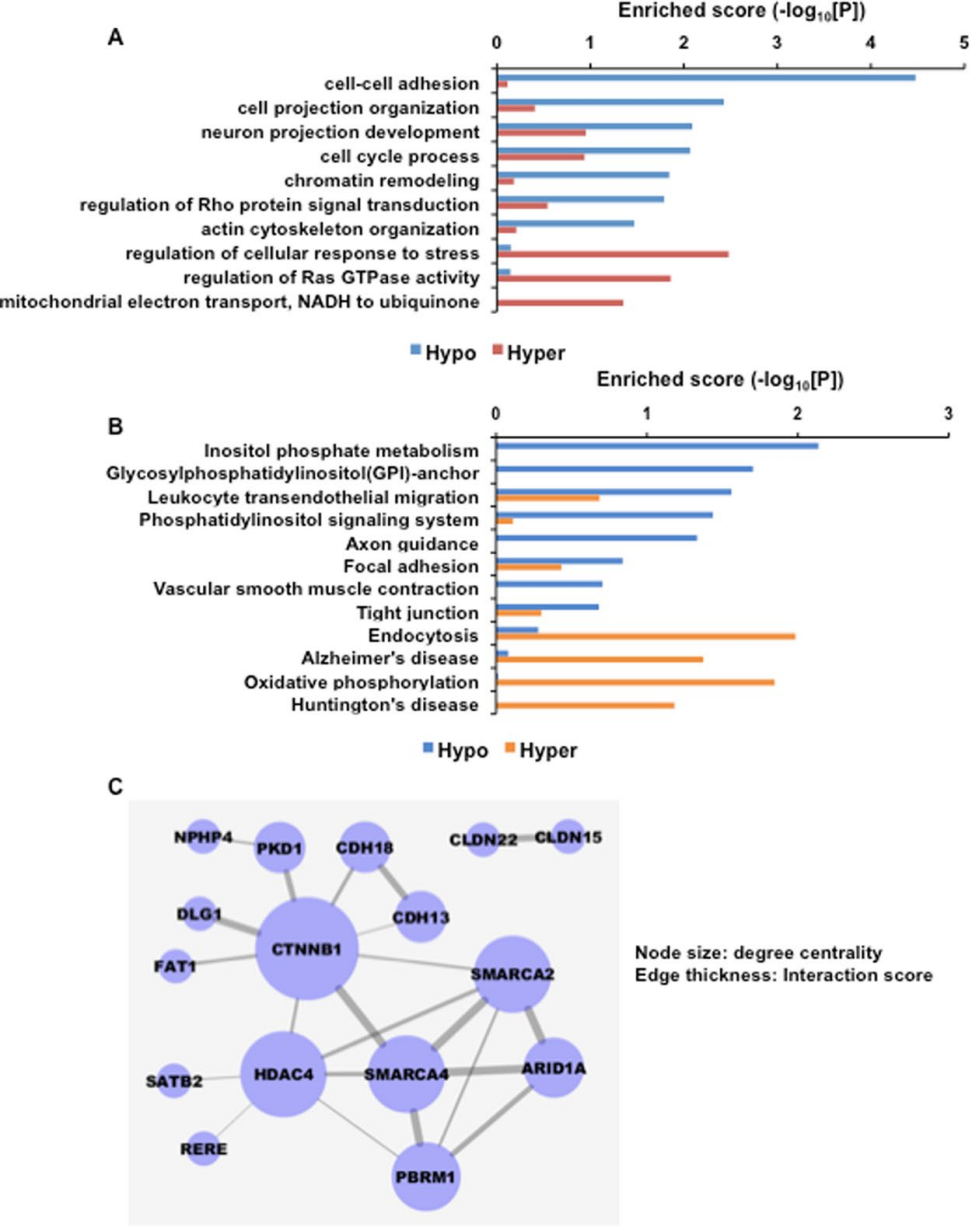
Enriched biology perturbed by α-oxoglutarate. (**A** and **B**) Enriched cellular processes (**A**) and KEGG pathways (**B**). Bar plot represents scores of enrichments by hyper- and hypo-methylated genes. (**C**) Network model describes cell-cell adhesion and chromatin remodeling genes that were hypo-methylated by α-OG treatment.

In order to explore the contribution of DNA methylation signatures in IC, we performed functional enrichment analysis and network modeling on the DMGs. Enriched cellular processes and biological pathways perturbed by α-OG treatment were identified by using DAVID software^25^. Hypo-methylated genes were enriched for cell-cell adhesion, cell projection organization, neuron projection development, cell cycle processes, chromatin remodeling, and Rho protein signal transduction regulation, while cellular responses to stress, Ras GTPase activity, and transport of NADH to uniquitinone in the mitochondrial electron transport chain were enriched for by hyper-methylated genes **(**Fig. 4A**)**. We also found that KEGG pathways. inositol phosphate metabolism, glycosylphosphatidylinositol-anchor biosynthesis, leukocyte trans-endothelial migration, phosphatidylinositol signaling were enriched for by hypo-methylated genes and endocytosis, Alzheimer’s disease, oxidative phosphorylation, Huntington’s disease were enriched for by hyper-methylated genes **(**Fig. 4B**)**. Table 1 shows the related DMGs for each of the enriched cellular processes.

**Table 1:** Lists of DMGs for each of the enriched cellular processes.

Term	Hypo-methylated genes	Hyper-methylated genes
cell-cell adhesion	PCDHGA9, PCDHGA8, PCDHGA7, PCDHGA6,PCDHGA5, PCDHGA4, PCDHGA3, PCDHGA2, CTNNB1, PCDHGA1, CLDN15, PCDHGB1, NPHP4, FAT1, COL6A2, PKD1, DLG1, PCDHGA12, PCDHGA10, PCDHGA11, PCDHGB7, CLDN22, PCDHGB6, PCDHGB3, CERCAM, PCDHGB2, PCDHGB5, PCDHGB4, CDH13, CDH18	CDK5R1, PARD3, EZR, CTNND2, PTPRT
cell projection organization	ADORA2A, PAX6, GRIN3A, TPM1, EPHB1, VCL, DFNB31, IGF1R, HOXA2, ATXN10, BCL11B, NUMB, DCLK1, PRKCA, MCF2, BAIAP2L1, BAIAP2, RXRA, NUP85, WWTR1, CDH13, TSC1, CYFIP1, MAPK8IP3, EFNA5, APBB2, APBB1, DST, NGF, MYH10	PPP1R9B, PARD6B, CDK5R1, PARD3, ILK, NUMB, SIAH1, LMX1A, FEZ1
neuron projection development	PRKCA, ADORA2A, MCF2, BAIAP2, RXRA, PAX6, GRIN3A, EPHB1, IGF1R, HOXA2, ATXN10, BCL11B, NUMB, MAPK8IP3, CYFIP1, EFNA5, APBB2, APBB1, DST, DCLK1, MYH10, NGF	PPP1R9B, PARD6B, CDK5R1, PARD3, ILK, NUMB, SIAH1, LMX1A, FEZ1
cell cycle process	MAD1L1, FZR1, KIF25, TSG101, POLA1, CETN1, CDC34, CDC16, TCF7L2, CTNNB1, PLAGL1, PSMF1, CUL2, RAD51L1, PSMD1, PBRM1, PKD1, HBP1, PSMD5, TCF3, ZW10, PSMD9, NFATC1, RAD52, WEE1, FOXN3, PPM1G, MNAT1, PPM1D, NOLC1, CUL4A, SYCP3, CDK2AP1, CDK11B, HORMAD1, CHFR, APBB2, APBB1, DST, MYH10	KIFC1, KIF22, DAXX, TCF7L2, CENPJ, DDIT3, PPP1R9B, KIF2C, CUL5, CUL4A, ILK, SKA2, PSMD4, MAPRE1, MAP9, DHCR24
chromatin remodeling	HDAC4, SATB2, SYCP3, PBRM1, ARID1A, SMARCA2, RERE, SMARCA4	BAZ1B, SUPT5H
regulation of Rho protein signal transduction	OBSCN, BCR, PLEKHG1, ABR, TSC1, PLEKHG7, MCF2, ARHGEF16, TRIO, ABRA, FARP1	PREX2, BCL6, MLST8, ARHGEF11
actin cytoskeleton organization	FMNL2, SHROOM3, TNXB, SSH1, CALD1, CYTH2, MYO9B, DAAM2, TPM1, ARHGAP26, NPHP4, SCIN, GRID2IP, DST, LCP1, MYH10, CDC42BPB, DLG1	PPP1R9B, EZR, BCL6, FHDC1, ARHGEF11
regulation of cellular response to stress	SH3RF1, MAP3K9, MAP3K10, MAPK8IP3, PKN1	FGF19, ERCC6, AIDA, POLH, EEF1E1, HIPK3, DAXX, SPP1
regulation of Ras GTPase activity	TBC1D2B, TSC1, TBC1D14, RABGAP1L, AGAP1	RABGAP1L, ASAP1, BCL6, EVI5L, MLST8, TBC1D20, AGAP2
mitochondrial electron transport, NADH to ubiquinone		NDUFS7, NDUFB10, NDUFA10, NDUFS1

Our previous study on RNA-sequencing analysis of a IC rat mimic model revealed significant association of cell adhesion and IC context^30^. In addition, gene expression changes of chromatin remodeling genes were identified in human bladder epithelial cells from patients with IC^30^. We thus focused on cell-cell adhesion and chromatin remodeling. To this end a network model describing the interactions of the hypo-methylated genes involved in cell-cell adhesion and chromatin remodeling were reconstructed with protein-protein interaction information from the STRING database^26^. This network model consisted of β-CATENIN (CTNNB1), PKD1 (polycystin 1), CDH18 (cadherin 18), SMARCA4 (SWI/SNF related, matrix associated, actin dependent regulator of chromatin, subfamily A, member 4), HDAC4 (histone deacetylase 4), ARID1A (AT-rich interactive domain 1A), PBRM1 (Polybromo-1, a component of the PBAF (Polybromo-associated-BRG1- or BRM-associated factors) chromatin remodeling complex) *et al*. Of note, CTNNB1 and SMARCA4 are valid candidates regulating cell adhesion and chromatin remodeling in this system **(**Fig. 4C).

### ARID1A expression significantly increased with α-OG treatment

**Figure 5:**
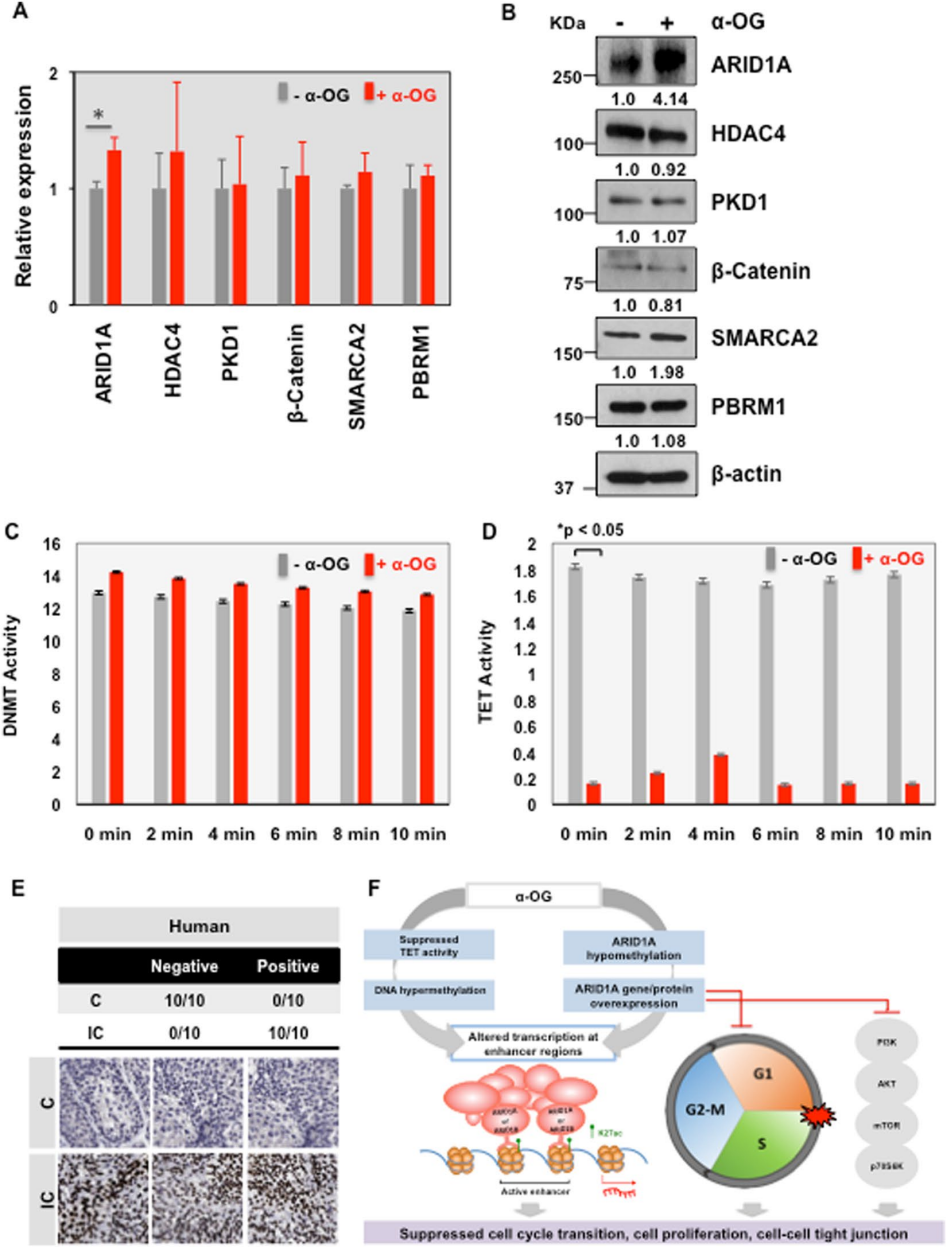
The expression levels of ARID1A were significantly increased in α-OG treated condition. (**A**) Quantification result from RT-PCR analysis to measure the gene expression levels of six candidate genes (ARID1A, HDAC4, PKD1, β-CATENIN, SMARCA2, and PBRM1) in the presence or absence of α-OG. *P < 0.05 (two-sided Student’s t-test). **(B)** Representative western blot show the levels of candidates (ARID1A, HDAC4, PKD1, β-CATENIN, SMARCA2, and PBRM1) and β-actin. **(C)** DNMT and **(D)** TET activity were measured in response to α-OG treatment. *P < 0.05 (two-sided Student’s t-test). **(E)** Representative Immunohistochemical stain showed ARID1A expression in bladder tissues obtained from IC patients (IC) and healthy donors (C). **(F)** A diagram showing the potential mechanism that redundant α-OG in urine may regulate bladder epithelial cells and contribute to suppression of cell proliferation.

ARID1A, HDAC4, PKD1, β-CATENIN, SMARCA2, and PBRM1 were suggested in our network model for IC and were selected for further validation. These genes were all hypo-methylated in response to α-OG treatment. To ascertain that α-OG treatment modifies the DNA methylome in TRT-HU1 cells, we next quantified the levels of DNA methylation and gene expression of the targeted candidate genes. Using quantitative RT-PCR analysis with customized primers **(**Supplementary Table 1**)**, we found the mRNA expression levels of all candidate genes as being slightly elevated. Gene expression of ARID1A was significantly increased in the α-OG treated condition (p = 0.018) **(**Fig. 5A). The Protein expression levels of ARID1A and SMARCA2 increased; however, those of HDAC4, PDK, β-CATENNIN, and PBRM1 did not **(**Fig. 5B).

### α-OG treatment altered the activity of ten-eleven translation (TET) hydroxylase but not DNA methyltransferase (DNMT)

To further elaborate on the effects of α-OG on epigenetic regulation in immortalized normal bladder epithelial cells, we measured the activity of two DNA methylation regulators, DNMT and TET, in the presence of α-OG **(**Fig. 5C**)**. In our experiments, α-OG treatment suppressed TET activity almost completely; however, there was no influence on DNMT activity **(**Fig. 5D).

On histological examination, we found that ARID1A expression greatly increased in IC patients. IHC analysis of bladder tissue samples obtained from 10 IC patients and 10 normal controls showed 100% positive staining in IC tissue for ARID1A, while none of the control tissue showed any staining **(**Fig. 5E). A hypothetical diagram suggesting epigenetic modulation on ARID1A by α-OG is shown in Fig. 5F.

## Discussion

Our previous reports provided evidence showing that bladder cell metabolism is altered in IC and suggested several urinary metabolites, including α-OG, as candidate biomarkers^16^. However, it is still unclear whether these metabolic biomarkers are biologically active in urine and how they contribute to bladder pathogenesis and homeostasis. The metabolic changes in the IC bladder are largely speculated to be associated with the microenvironment, resulting in altered oxygen states and metabolites. As products of this perturbed metabolism, these metabolites may be what are mediating the epigenetic alteration seen in the IC bladder. Here, our experimental results demonstrate that a novel IC biomarker, α-OG, directly reduces TET activity. α-OG also simultaneously causes DNA hypo-methylation of ARID1A and cell cycle transition arrest in immortalized normal bladder epithelial cells (Fig. 5F). These findings are consistent with clinical observations in patients; one characteristic of IC is thin bladder epithelium layers^31,32^. Previous mechanistic studies have shown that bladder epithelial cells derived from IC patients have suppressed proliferation and cell cycle arrest^31,33^. Based on these previous observations and our findings, α-OG may not only be a novel urinary metabolite associated with IC, but may also have an active biological function.

Altering the methylation status of DNA promoters and enhancers are widely linked to reductions in gene transcription. There are two main processes that orchestrate DNA methylation of key epigenetic regulators; these involve 5-methylcytosine (5mC) and 5-hydroxymethylcytosine (5hmC), which are located on the CpG islands of gene promoters and enhancers. Currently, the most well-studied process of covalent DNA modification is methylation through the addition of methyl groups to produce 5mC. Active DNA methyltransferases, such as DNMT1 and DNMT3A/B, use S-adenosylmethionine as methyl donors and are required for establishing and maintaining DNA methylation patterns. DNA demethylation is another important epigenetic mechanism and is mediated by TET, which converts 5mC to 5hmC and further to 5-carboxylcytosine (5-caC) through its hydroxylase activity.

In the context of IC, perturbed epigenetic architectures, such as DNA methylation and demethylation, in the bladder epithelium have not been carefully investigated. However, given that our encouraging data shows suppressed TET activity as a potential IC biomarker, it may be clinically significant to elaborate on the potential role of TET and its targets as diagnostic markers and/or therapeutic targets. It is not known how these changes in TET and 5hmC profiles are related to IC and how TET can be reactivated. What is known so far is that the TET family (TET1, TET2, and TET3) plays an important role in hydroxymethylating and demethylating 5mC in IC. It is still unclear how these changes in TET and 5hmC profiles are related to IC and how TET can be reactivated. Unfortunately, due to the lack of commercially available TET activators, we could not perform any tests to see if TET stimulation can reverse bladder dysfunction.

Our results also suggest the possible physiological role of ARID1A in maintaining normal homeostasis in the bladder epithelium through controlling chromatin remodeling. ARID1A and SMARCA2, both are subunits of the SWI/SNF chromatin-remodeling complex, were suggested as key players of α-OG signaling pathway in our network model. Although modest, the expression of SMARCA2 also increased upon α-OG treatment (Fig. 5B). This finding is consistent with previously published microarray analyses of IC primary culture cells and controls. The gene expression of both IC patient-derived primary culture cells and APF (anti-proliferative factor, an urinary IC biomarker)-treated cells suggested a less proliferative phenotype, with increased expression of SMARC2^34^.

ARID1A sustains chromatin accessibility through H3K27me3 and H3k27ac in the enhancer regions^35,36^. ARID1A is also a well-known bona fide tumor suppressor that is frequently inactivated by mutations or reduced in expression due to promoter hyper-methylation^37^. Along with p21, cyclins, and E2F-responsive genes, ARID1A is essential for normal cell cycle arrest; thus, inactivation of ARID1A results in tumor transformation, metastasis, and/or drug resistance^38,39^. However, the biological function of ARID1A is context dependent. In bladder cancer, increased ARID1A expression is generally correlated with higher tumor grade; however, there is some variation based on the cancer type^40,41^. Detailed information regarding the mechanism and physiological roles of ARID1A and its co-factors has been lacking. Based on our study, we propose that ARID1A can be regulated through α-OG, contributing to an impaired cell proliferation in IC. We believe that additional attempts to validate the epigenetic regulation of α-OG on ARID1A in clinical samples may provide novel insight into the etiology of IC and identify metabolites that can serve as IC biomarkers for clinical application.

In summary, evidence from our EPIC DNA methylation profiling suggests that α-OG, a potential IC biomarker candidate, regulates ARID1A epigenetically. Modification of the promoter for ARID1A contributes to increased expression at both the mRNA and protein levels, leading to suppressed proliferation in the bladder epithelial cells. These findings indicate that urinary metabolites in the bladder can lead to epigenetic reprogramming. Given our observations, we may have defined the epigenetic regulatory mechanisms of ARID1A and TET activity in the anti-proliferative axis in immortalized normal bladder epithelial cells.
