# Author Correction: Alpha-oxoglutarate inhibits the proliferation of immortalized normal bladder epithelial cells via an epigenetic switch involving ARID1A

**DOI:** 10.1038/s41598-018-24827-9

**Published:** 2018-04-16

**Authors:** Muhammad Shahid, Nicole Gull, Austin Yeon, Eunho Cho, Jooeun Bae, Hyun Seok Yoon, Sungyong You, Hana Yoon, Minjung Kim, Benjamin P. Berman, Jayoung Kim

**Affiliations:** 1 Departments of Surgery and Biomedical Sciences, Cedars-Sinai Medical Center, Los Angeles, CA USA; 2 Center for Bioinformatics and Functional Genomics, Department of Biomedical Sciences, Cedars-Sinai Medical Center, Los Angeles, California, USA; 3 University of California Los Angeles, Los Angeles, CA USA; 4 Department of Urology, School of Medicine, Ewha Womans University, Seoul, Republic of Korea; 5 Department of Molecular Oncology, Moffitt Cancer Center, Tampa, Florida USA; 6 Samuel Oschin Comprehensive Cancer Institute, Cedars-Sinai Medical Center, Los Angeles, CA USA; 7 Department of Urology, Ga Cheon University College of Medicine, Incheon, Republic of Korea

Correction to: *Scientific Reports* 10.1038/s41598-018-22771-2, published online 14 March 2018

In the original version of this Article, Muhammad Shahid and Nicole Gull were omitted as equally contributing authors. This has now been corrected in the PDF and HTML versions of the paper.

